# Exploring the Association Between Structural Racism and Mental Health: Geospatial and Machine Learning Analysis

**DOI:** 10.2196/52691

**Published:** 2024-05-03

**Authors:** Fahimeh Mohebbi, Amir Masoud Forati, Lucas Torres, Terri A deRoon-Cassini, Jennifer Harris, Carissa W Tomas, John R Mantsch, Rina Ghose

**Affiliations:** 1 College of Engineering and Applied Science University of Wisconsin-Milwaukee Milwaukee, WI United States; 2 Department of Medicine University of Wisconsin-Madison Madison, WI United States; 3 Department of Psychology Marquette University Milwaukee, WI United States; 4 Division of Trauma & Acute Care Surgery Department of Surgery Medical College of Wisconsin Milwaukee, WI United States; 5 Community Relations-Social Development Commission Milwaukee, WI United States; 6 Division of Epidemiology Institute for Health and Equity Medical College of Wisconsin Milwaukee, WI United States; 7 Department of Pharmacology & Toxicology Medical College of Wisconsin Milwaukee, WI United States

**Keywords:** machine learning, geospatial, racial disparities, social determinant of health, structural racism, mental health, health disparities, deep learning

## Abstract

**Background:**

Structural racism produces mental health disparities. While studies have examined the impact of individual factors such as poverty and education, the collective contribution of these elements, as manifestations of structural racism, has been less explored. Milwaukee County, Wisconsin, with its racial and socioeconomic diversity, provides a unique context for this multifactorial investigation.

**Objective:**

This research aimed to delineate the association between structural racism and mental health disparities in Milwaukee County, using a combination of geospatial and deep learning techniques. We used secondary data sets where all data were aggregated and anonymized before being released by federal agencies.

**Methods:**

We compiled 217 georeferenced explanatory variables across domains, initially deliberately excluding race-based factors to focus on nonracial determinants. This approach was designed to reveal the underlying patterns of risk factors contributing to poor mental health, subsequently reintegrating race to assess the effects of racism quantitatively. The variable selection combined tree-based methods (random forest) and conventional techniques, supported by variance inflation factor and Pearson correlation analysis for multicollinearity mitigation. The geographically weighted random forest model was used to investigate spatial heterogeneity and dependence. Self-organizing maps, combined with K-means clustering, were used to analyze data from Milwaukee communities, focusing on quantifying the impact of structural racism on the prevalence of poor mental health.

**Results:**

While 12 influential factors collectively accounted for 95.11% of the variability in mental health across communities, the top 6 factors—smoking, poverty, insufficient sleep, lack of health insurance, employment, and age—were particularly impactful. Predominantly, African American neighborhoods were disproportionately affected, which is 2.23 times more likely to encounter high-risk clusters for poor mental health.

**Conclusions:**

The findings demonstrate that structural racism shapes mental health disparities, with Black community members disproportionately impacted. The multifaceted methodological approach underscores the value of integrating geospatial analysis and deep learning to understand complex social determinants of mental health. These insights highlight the need for targeted interventions, addressing both individual and systemic factors to mitigate mental health disparities rooted in structural racism.

## Introduction

Structural Racism and Discrimination (SRD) is a fundamental determinant of health disparities and poor health outcomes among historically marginalized communities in the United States [1]. SRD refers to systemic or institutional racism and societal norms that constrain the chances, resources, influence, and welfare of people and communities due to their racial and ethnic background and other characteristics. Racial segregation in urban centers is an outcome of SRD-driven policy inequalities such as discriminatory mortgage lending practices (redlining), confining Black Americans to central city neighborhoods, which became sites of concentrated poverty and heightened inequities. Residents of segregated neighborhoods experience disproportionate exposure, susceptibility, and vulnerability to economic and social inequality, environmental pollution, toxic substances, and unsafe conditions, thereby affecting individual health conditions, health practices, and access to health care services [2]. Further, neighborhood-level racial and ethnic segregation determines and limits access to educational, employment, and health-related resources [1]. Studies have emphasized the significance of neighborhood segregation on health inequity [3].

Past research has focused on the relationship between interpersonal discrimination and health; however, SRD is likely to have broad downstream effects on psychological, biological, physiological, and behavioral processes [4]. Mental health is of particular importance, especially since the start of the COVID-19 pandemic, where estimates of pooled prevalence of depression are 7 times higher than expected [5], with minority Americans experiencing more severe and chronic symptoms across time [6]. As individuals are embedded within larger systems of influence, it is essential to understand the relationship between SRD and mental health at a community level [5]. The socioecological model of health provides a framework to examine how individual health and behavior are impacted by interpersonal, neighborhood, and societal factors [7]. The National Institute on Minority Health and Health Disparities (NIMHD) has encouraged a place-based approach, as “relationships between SRD and physical/mental health are influenced by numerous place-based factors… individual-level factors (i.e., health-related behaviors, ways of coping) are understood best within the context of the lived environment and structural policies that perpetuate inequities” [1].

To examine the contribution of SRD to inequalities, various measures have been used including racial residential segregation [8]. However, as no domain of structural racism operates in isolation, multiple index measures of SRD have been created and applied. Dougherty et al [9] developed a structural racism index measure by combining 7 measures of SRD: housing dissimilarity index, school dissimilarity index, high school graduation ratio, incarceration ratio, poverty ratio, primary care ratio, and ambulatory care ratio. Still, there are no consistent, agreed-upon relevant content domains of structural racism [10]. Instead of selecting 1 approach or developing yet another index, we examined mental health disparities without reference to race to determine whether communities experiencing SRD can be identified using an unsupervised retrospective approach.

This study used the NIMHD framework to explore the relationship between SRD and mental health. By using machine learning algorithms with geodata science, we conducted spatial modeling and geovisualization to investigate how location-based factors, indicative of SRD, impact mental and physical health. This approach allows for a statistical analysis of these dynamics over time and space, offering a comprehensive analysis of the effects of place on health outcomes [11]. Data were analyzed at multiple geographic scales—county, city, and census tracts. Understanding mental health at the community level is important, given the complex intersectionality of factors that promote or hinder health in the United States [12].

Our study site is Milwaukee County (population of 918,661), the most populous county in Wisconsin, with a racially diverse population of Black (n=241,608, 26.3%), Hispanic or Latino (n=143,311, 15.6%), and White origin (n=541,091, 58.9%) [13]. The county includes the hypersegregated city of Milwaukee (population 563,305), where redlining confined the African American population to its central city neighborhoods. Devastated by deindustrialization and disinvestments, these neighborhoods exhibit concentrated urban poverty, heightened sociospatial inequalities, and dramatic health disparities [14], where Black residents experience a poverty rate 5 times higher than that of White residents and White residents outlive Black residents by almost 14 years [13]. Acknowledging the damaging legacy of SRD on health, Milwaukee County was among the first jurisdictions in the United States to declare racism as a public health crisis in 2019, with 170 jurisdictions following its suit.

## Methods

### Overview

To identify geospatial determinants of health across behavioral indicators, built environment, sociocultural environment, and health care (based on the NIMHD framework), georeferenced data sets were acquired from the United States Census Bureau. The United States Census Bureau anonymizes and deidentifies data before releasing them to the public. Detailed demographic data can be obtained only at the Census Tract level (each tract comprises 4000 residents) and not at an individual level. In accordance with census policies, we analyzed all data at the census tract level, and no individual-level data have been obtained or used. Selected variables (eg, age, gender, population, race, ethnicity, marital status, educational attainment, educational enrollment, employment, neighborhood stability, and poverty) were compiled into a data set of 217 explanatory variables. Notably, race-based factors were deliberately withheld during the initial stages of our unsupervised machine learning analysis. This approach allowed the model to operate without the assumption that racial disparities significantly influence mental health outcomes. It was only in the final stage of the analysis that race and ethnicity variables were incorporated, providing an opportunity to observe whether the emerging patterns of poor mental health prevalence correlated with racial factors. All variables were joined to the administrative boundary shapefile of Milwaukee County census tracts collected from the TIGER/Line database11 (using ArcGIS Desktop 10.7; Environmental Systems Research Institute).

### Variable Selection Procedure

#### Overview

Variable selection minimizes the number of predictors in quantitative models to improve efficiency and reduce complexity. Public health research commonly uses conventional techniques such as subject matter expert selection and regression-based stepwise selection. However, tree-based methods such as random forest can handle nonlinear, nonparametric relationships and provide more robust results [15].

Extreme multicollinearity can cause parameter estimate instability, unintuitive parameter signs, high *R*^2^ diagnostics despite few or no significant parameters, and inflated standard errors of the parameter estimates [16]. To avoid this, variance inflation factor (VIF) and Pearson correlation analysis are used to detect multicollinearity and select the most uncorrelated variables for the random forest variable selection model. The variable selection method [17] implemented in the Variable Selection Using Random Forest (VSURF) R package (R Foundation for Statistical Computing) yields the best results compared with other variable selection and baseline methods [18]. The method ranks variables by their importance, eliminating the least important ones and constructing a sequence of random forest models. Eventually, variables of the most optimized model are selected.

#### Geographically Weighted Random Forest

Spatial modeling enables the examination of how variables behave across geographical space to identify spatial heterogeneity and dependence. Local spatial modeling offers a more informed approach to understanding complex phenomena compared with conventional global approaches [19]. The geographically weighted random forest (GWRF), a localized model, is ideal for conducting public health research. However, GWRF has not been used to examine the variability in relationships between place-based risk factors and the prevalence of poor mental health. This study used GWRF to investigate nonstationarity and localized associations between risk factors and the prevalence of poor mental health.

Local feature importance in GWRF measures how much each feature contributes to the accuracy of the model within a specific region, calculated by the increase in mean squared error or the decrease in node impurities averaged over all trees in the model. These measures are derived from the out-of-bag (OOB) error, which is a measure of the model’s performance on data not used during training [20].

#### Self-Organizing Maps

Self-organizing maps (SOMs) are unsupervised artificial “neural” networks that create a 2D space topographic map of a data set. “Neurons,” or relational clusters, are organized to preserve their context or neighborhood, and SOMs use closeness or neighborhood function to display input space properties. We implemented a SOM model to explore a data set containing 296 census tracts in Milwaukee County×12 determinant factors of poor mental health. The SOM was trained on this data set using a 5×5 hexagonal grid, selected for its ability to create a more comprehensive and representative map of the data. After training the SOM, we delved deeper into the clusters formed on the map by combining the SOM with k-means clustering, an algorithm known for its efficiency in partitioning data into distinct groups or clusters. We determined the optimal number of clusters for our data set by evaluating the “within-cluster sums of squares” and the “average silhouette” statistics [21].

### Ethical Considerations

This study uses anonymized and deidentified publicly available data sets that were released by federal or state agencies for public consumption. All publicly available government data are aggregated at the census tract level, where each tract is composed of 4000 individuals. We received full approval from the Medical College of Wisconsin institutional review board with exemption from oversight by Code of Federal Regulations 46.104 (d) 4, as we conducted secondary analyses of publicly available anonymized and deidentified data sets that do not contain any personally identifiable information and do not pose any risk to ethical violations. Given that the project did not involve direct contact with subjects, an informed consent process was not required and no compensation was provided. Moreover, the project did not require a waiver of HIPAA (Health Insurance Portability and Accountability Act) authorization as outlined in 45 Code of Federal Regulations 164.514(e) because it used a Limited Data Set.

## Results

### Input Factor Selection

We compiled a database of 217 geospatial determinants of health across behavioral indicators, built environment, sociocultural environment, and health care to analyze the relationship between place-based factors and mental health. Next, we excluded all race-based factors and used the remaining 184 variables to identify highly colinear variables, VIF, and Pearson correlation analysis was applied (VIF threshold=7.5; Pearson correlation threshold=0.75). As a result, 105 uncorrelated factors were selected to be used as the input for the variable selection (VSURF) procedure.

### User Statistics

The VSURF procedure was applied to the 105 place-based factors to find a sufficiently parsimonious set of significant indicators of poor community mental health (ie, the percentage of adults who stated that their mental health was not good 14 or more days in the past month) and measures of SRD. The VSURF selected 98 variables at the thresholding step, then 24 variables at the interpretation step, and 12 variables at the prediction step. The most important place-based factors of mental health were adults who smoke, insufficient sleep, adults without health insurance, adults who are obese, adults who are sedentary, marriage rate, people living below the poverty level, childhood opportunity index score, median age, homeownership, full-time employment, and educational attainment (all variables are percentages except for Age and Childhood Opportunity Index score). These variables account for 95.11% of the variability in poor mental health in communities, with a mean of squared residuals of 0.74.

**Figure 1:**
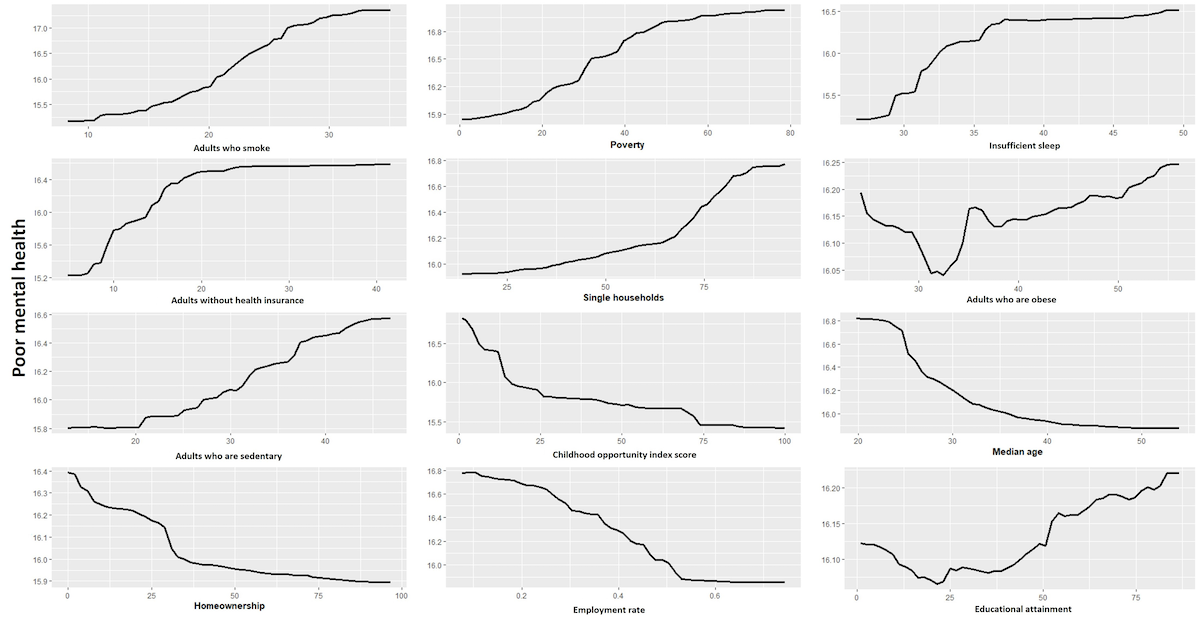
Partial dependence plots illustrate the marginal effect of each of the 12 selected predictor variables on the prevalence of poor mental health. The Y-axis in each plot indicates the prevalence of poor mental health outcomes, quantified as a percentage, and the X-axes represent the value of each predictive variable. The curve in each partial dependence plot shows the relationship between the predictor variable and the prevalence of poor mental health outcomes. An ascending curve implies that an increase in the predictor variable corresponds with an increase in poor mental health outcomes, while a descending curve implies the opposite.

Partial dependence plots depicting the relationship of each of the 12 predictive variables for poor mental health were constructed. Figure 1 illustrates the correlation between the selected 12 variables and poor mental health. The results of the partial dependence plot approach showed that the relationship among the prevalence of smoking, poverty, insufficient sleep, lack of health insurance, single households, sedentariness, and poor mental health was positive. In contrast, the childhood opportunity index, median age, homeownership, and employment rate exhibit negative relationships with poor mental health. Notably, obesity and educational attainment did not follow linear trends in relation to poor mental health, illustrating the complexity of these factors. The largest difference makers were the prevalence of smoking, lack of insurance, poverty, insufficient sleep, employment, and age.

### GWRF Implementation

**Figure 2:**
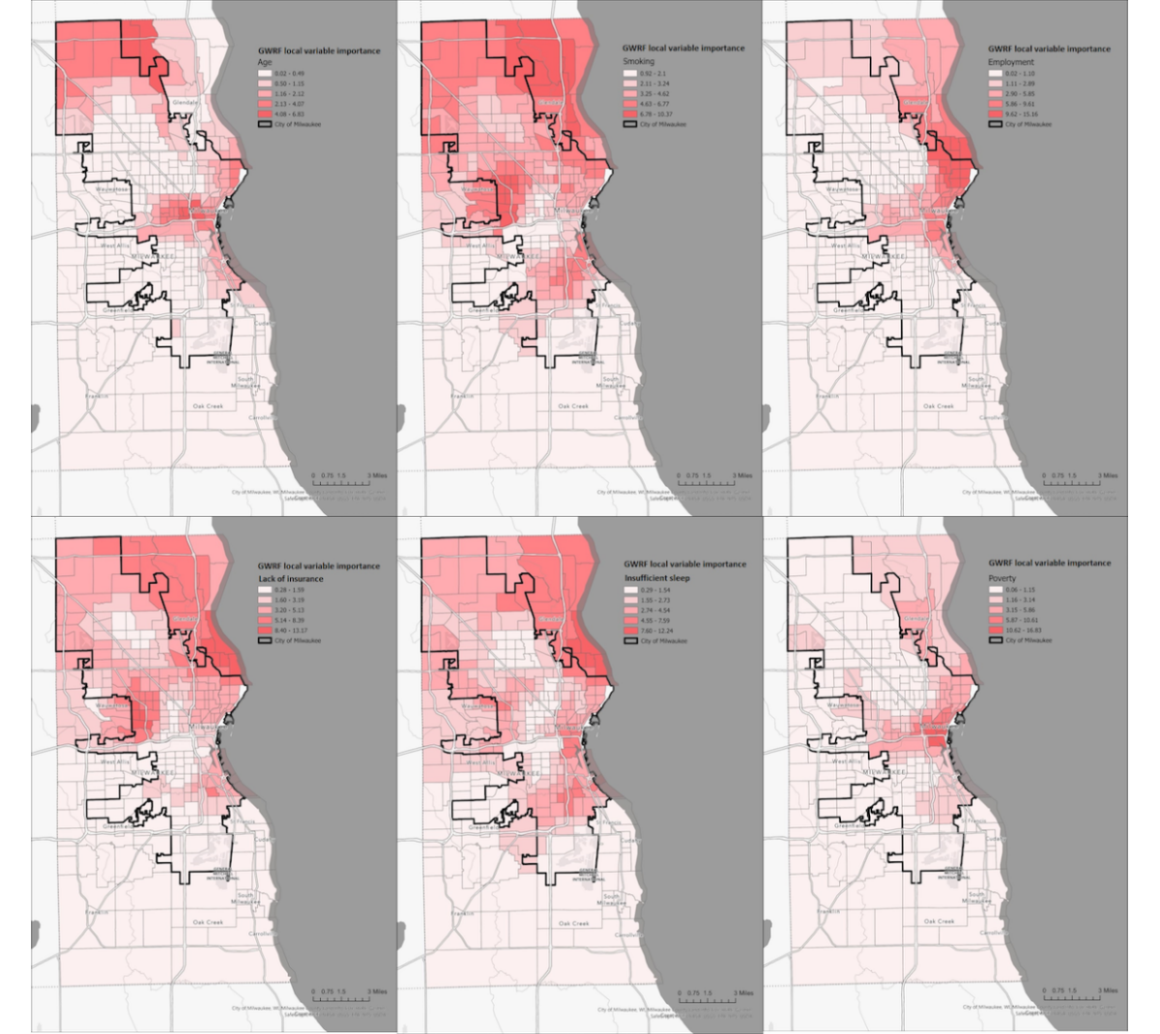
Geographic variation in place-based risk factors impacting mental health. Maps represent a visualization of the localized influences of 6 place-based risk factors—prevalence of smoking, lack of health insurance, poverty level, insufficient sleep, employment status, and median age—on predictions of poor mental health. Visualization is based on the findings of the GWRF model. Each risk factor is represented in a separate map, with color gradients signifying areas of varying impact on mental health. Darker shades indicate regions where a risk factor significantly correlates with poor mental health, while lighter shades correspond to weaker correlations. These visual representations underscore the spatial heterogeneity of mental health determinants and the significance of targeted interventions. GWRF: geographically weighted random forest. For a higher-resolution version of this figure, see Multimedia Appendix 1.

Next, we used GWRF to investigate and visualize local associations between poor mental health prevalence and the 12 identified place-based risk factors at multiple scales—the county, the city, and census tract levels. Six place-based risk factors (prevalence of smoking, lack of insurance, poverty, insufficient sleep, employment, and age) were identified as having a spatially heterogeneous impact. We trained the GWRF with 45 nearest neighbors (census tracts) with bootstrapped 5000 “ntrees” and 4 “mtry” in each tree. It yielded a local model with an *R*^2^ square (OOB) of 94.91%, mean squared error (OOB) of 0.051, and Akaike Information Criterion (OOB) of –856.447. Spatial variation of the local contribution of the 6 risk factors to the prediction of poor mental health is shown in Figure 2.

### SOMs Implementation

**Figure 3:**
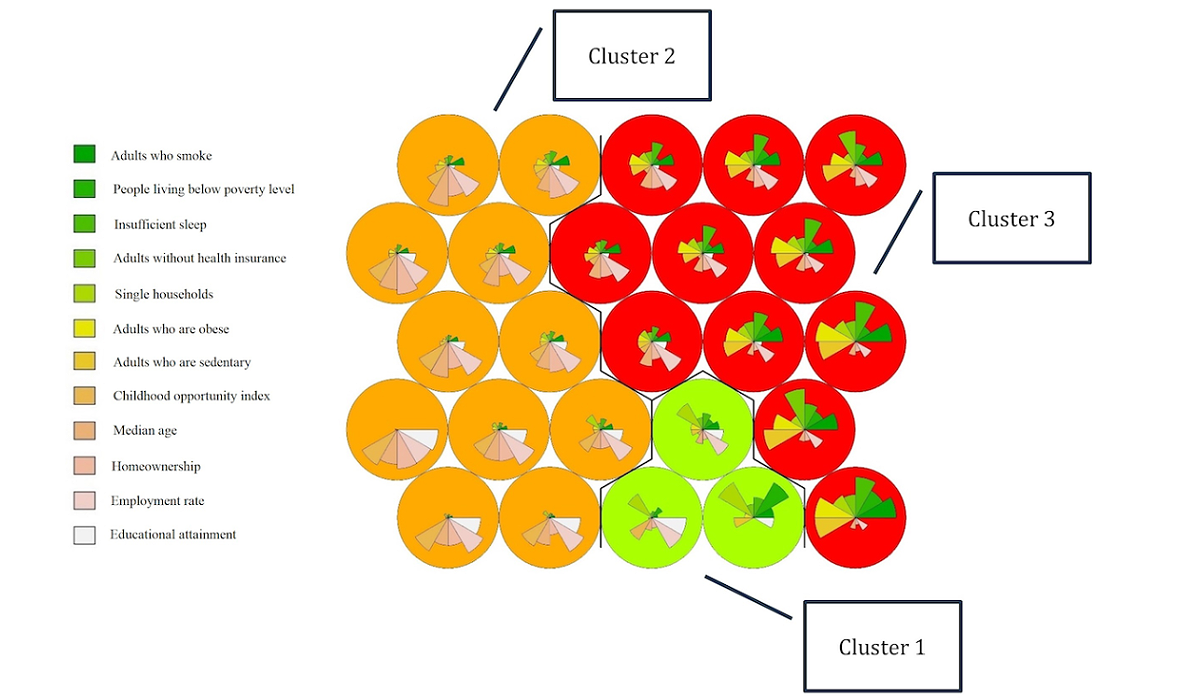
Integrated self-organizing map and k-means showing the 3 clusters of census tracts in Milwaukee County based on similarities among the 12 mental health risk factors. The differences between clusters are maximized and within clusters are minimized. The size or area of each wedge reflects its proportional “influence” on mental health in the corresponding census track grouping.

A SOM approach was used to determine impactful clusters of the 12 most significant place-based factors related to mental health. Census tracts were grouped based on similarities in these mental health–related factors. The SOM was integrated with k-means clustering to further delineate and group census tracts based on their similarity. The original 25 SOM grid codes were condensed into 3 clusters, depicted in Figure 3. Each cluster represents a collection of census tracts displaying similar patterns or characteristics across the 12 mental health–related factors. Clusters 1, 2, and 3 differ markedly in their composition, each corresponding to varying levels of mental health risk. Although a more detailed understanding of each cluster necessitates additional data, particularly in terms of specific demographic, socioeconomic, and environmental variables that may influence these risk levels, these clusters can be generally categorized as representing high (cluster 3), moderate (cluster 2), and low (cluster 1) risk for poor mental health. This categorization is based on the aggregated mental health factors within each cluster. Deeper analysis with more comprehensive data could reveal more nuanced distinctions and cluster-specific contributing factors.

**Figure 4:**
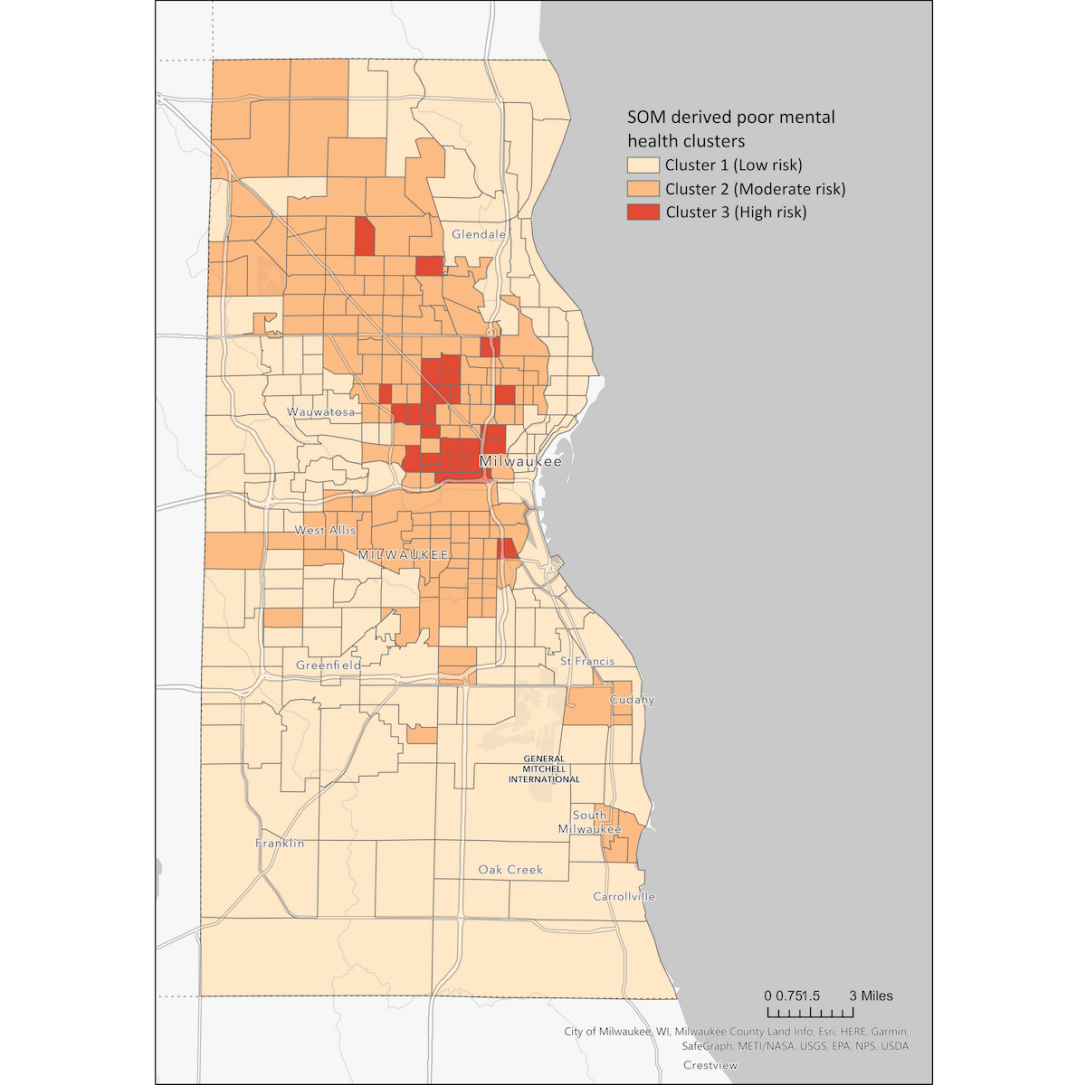
Spatial distribution of mental health risk clusters in Milwaukee County. This map presents the locations of communities that comprise the 3 risk clusters—low, moderate, and high (clusters 1-3)—identified based on 12 key place-based mental health factors. SOM: self-organizing map.

Figure 4 represents the geospatial distribution of the 3 mental health risk clusters across Milwaukee County. Classification into low-, moderate-, and high-risk labels refers to the collective intensity of the 12 key place-based mental health factors within each cluster. For instance, the “high-risk” label indicates the prevalence of higher levels of risk factors (ie, smoking prevalence, lack of health insurance, and poverty) known to contribute to poor mental health but does not necessarily mean that all individuals in this cluster have poor mental health.

To examine racial disparities within community clusters, we calculated a disproportionality index, the ratio of the percentage of a specific racial demographic within each cluster compared with that within a base population. Within the low-risk group (cluster 1), the disproportionality index is 1.5 for the White population, indicating an overrepresentation; the Black and Hispanic populations are underrepresented with indices of 0.21 and 0.6, respectively.

In the moderate risk group (cluster 2), the White population is underrepresented, with an index of 0.6; the Black and Hispanic populations are overrepresented, with indices of 1.52 and 1.51, respectively. The most significant disparity was found in the high-risk group (cluster 3), where the Black population’s disproportionality index was 2.23, and the White and Hispanic populations were underrepresented with indices of 0.34 and 0.51, respectively.

The findings of our unsupervised approach demonstrate significant mental health disparities that align with the racial demographics in Milwaukee County, despite our exclusion of race-related factors. The overrepresentation of Black populations in the high-risk cluster combined with the overrepresentation of the White population in the low-risk cluster indicates that disparities observed across clusters are not isolated occurrences but rather are indicative of underlying socioeconomic disparities likely caused by structural racism and discrimination. Notably, while Hispanic community members were underrepresented in the low-risk cluster 1 and overrepresented in the moderate-risk cluster 2, they were underrepresented in the high-risk cluster 3. This highlights that there are differences across racial minority demographics that influence mental health. For example, differences in upward financial mobility have been identified between Black and Hispanic communities [22].

The spatial dependency of risk areas was investigated to discover the locationality of poor mental health areas. To examine the degree of similarity, the Global Moran I index was calculated to measure the degree of similarity between an area and its neighboring areas. The calculated Moran Index is 0.53 with a *z* score of 21.63, indicating that there is <1% likelihood that the clustering of poor mental health risk was the result of random chance. The findings, displayed in Figure 4, show that clusters 2 and 3 are primarily located in central city neighborhoods of Milwaukee County, aligning with predominantly Black communities in the central and north side of the city and with the Hispanic community in Milwaukee’s south side.

Although generated without incorporating location as a factor, Figure 4 demonstrates spatial autocorrelation, signifying the presence of spatial dependency in mental health outcomes. This suggests that mental health issues are not randomly distributed but rather display geographically linked patterns. Spatial dependency confirms the influence of location or spatial context on mental health. Our results align with previous findings, further demonstrating that mental health outcomes are partly a product of spatially located phenomena such as socioeconomic conditions, access to health care, environmental factors, and SRD. The observations reinforce the assertion that conventional global models are not ideal for examining complex phenomena such as community mental health or SRD [19,23]. Local spatial modeling offers more precise outcomes when processes are affected by both their geographical location and the fluctuating conditions of underlying variables across different times and locations.

## Discussion

### Principal Findings

To address the complex issue of SRD and its impact on mental health disparities, our study used a 5-step process. First, we compiled a comprehensive database of place-based explanatory factors, also known as “geospatial determinants of health,” at the census tract scale [24]. Second, we deliberately eliminated all race-based factors, an unprecedented approach to assess whether SRD effects on mental health disparities could be examined without directly referencing race. Third, we conducted a variable selection process, identifying the most defining place-based characteristics that shape the mental health conditions of an affected community. Fourth, using SOMs, we clustered census tracts based on mental health risk factor similarity. Finally, we investigated the racial composition, demographic characteristics, and spatial dependency of risk areas to shed light on racial disparities, confirmed SRD, and illuminated the locationality of mental health and SRD (Table 1).

Our study introduces a novel approach to analyzing mental health disparities and their structural underpinnings, diverging from traditional models. By integrating advanced geospatial and machine learning techniques, it provides a more granular, community-specific insight into these disparities. This method contrasts with conventional approaches that may not fully capture the intricate, locality-specific interactions of factors impacting mental health [8]. In line with the study by Groos et al [8] based on the exploration of structural racism quantification methods, this study exemplifies the shift toward more sophisticated, nuanced methodologies in understanding the complex effects of SRD on health outcomes.

**Table 1:** Racial composition and demographic and socioeconomic characteristics of mental health risk areas^a^.

Cluster (n)	White, n (%)	Black, n (%)	Hispanic, n (%)	Homeownership, n (%)	Poverty, n (%)	Median household income (US $)	Employment, n (%)	Educational attainment (bachelor), n (%)	Poor mental health, n (%)	Poor physical health, n (%)	Incarceration, n (%)
1 (488,282)	408,839 (83.73)	31,885 (6.53)	42,822 (8.77)	282,715 (57.9)	24,463 (5.01)	71,913	285,547 (58.48)	152,832 (31.30)	93,701 (19.19)	106,543 (21.82)	11,182 (2.29)
2 (409.305)	144,853 (35.39)	191,145 (46.7)	90,334 (22.07)	138,550 (33.85)	90,538 (22.12)	39,801	179,808 (43.93)	41,012 (10.02)	97,374 (23.79)	107,115 (26.17)	21,161 (5.17)
3 (56,622)	10,753 (18.99)	38,718 (68.38)	4286 (7.57)	7474 (13.2)	27,320 (48.25)	20,889	15,616 (27.58)	3397 (6)	16,664 (29.43)	17,190 (30.36)	3907 (6.9)
County (954,209)	531,208 (55.67)	292,370 (30.64)	139,601 (14.63)	407,638 (42.72)	161,548 (16.93)	52,485	466,704 (48.91)	183,781 (19.26)	212,407 (22.26)	234,831 (24.61)	38,455 (4.03)

^a^With the exception of median household income, all data represent a percentage of the population in the census tracts that comprise each cluster. Data sets were sourced from the Centers for Disease Control and Prevention’s 500 Cities Project and the United States Census Bureau.

### Factors That Predict Poor Mental Health

Several relationships were identified, demonstrating the complex determinants of mental health in different contexts. A link between smoking prevalence and mental health was noted, revealing regional variation and reflecting broader patterns in mental health research. While the percentage of adults reporting poor mental health increased with smoking [25,26], the trend flattened at a prevalence of 17%. Smoking was more significantly associated with poor mental health in central city neighborhoods and northern suburbs of Milwaukee County but had a lower effect in the south side.

Access to health insurance is vital for mental health resource use. Our analysis emphasizes the necessity of removing structural barriers to mental health such as insurance coverage [27,28]. We find a strong correlation between poor mental health and the percentage of adults without health insurance. However, the trend flattens at a 16.6% prevalence of poor mental health, indicating that when more than 20% of a community lacks health insurance, other factors may contribute to poor mental health. The lack of health insurance affects mental health more significantly in central and northern neighborhoods of Milwaukee City.

The connection between poverty and mental health not only emphasizes the importance of a comprehensive investigation into policy, poverty, inequality, and mental health outcomes but also highlights the multifaceted nature of mental health challenges [29]. Our study shows that the relationship is complex and plateaus when more than 50% of community lives below the poverty level. Overall, poverty is a stronger defining characteristic of poor mental health in the central neighborhoods of Milwaukee.

Sleep deprivation and unemployment were identified as significant predictors of mental health [30-32]; sleep deprivation is a stronger predictor of poor mental health in central city neighborhoods and northern parts of Milwaukee County but is less of a predictor in southern communities. Our study shows that community employment has a strong negative association with poor mental health, but the relationship plateaus after 52% employment prevalence. Employment is more predictive of poor mental health in both the central city neighborhoods and the affluent shoreline areas of Milwaukee County.

Age is a determinant of mental health. Adult mental health diagnoses often begin in adolescence, with approximately half of all adult mental health disorders emerging in the teenage years [33]. The median onset age ranges from 8 to 35 years, and increased age is associated with better mental health [34]. Chen et al [35] found that the relationship between census tracts’ median age and prevalence of poor mental health follows a negative curvilinear trend, consistent with a large-scale meta-analysis of 192 epidemiological studies. Our results demonstrate that poor mental health in the central city neighborhoods of Milwaukee County is more significantly associated with age than in other parts of the county, indicating that the influence of age on mental health varies locally.

Although high-risk clusters showed high spatial alignment with socioeconomically disadvantaged communities in Milwaukee, several individual factors were strong predictors of mental health in more affluent and socioeconomically secure areas (eg, in Milwaukee’s lakefront communities). These include unemployment, lack of insurance, sleep deprivation, and smoking. These observations suggest that while these communities are not locations of high-risk clusters, mental health associations are evident in subgroups of community members.

### Geographical Disparities in Mental Health Challenges

Insights gained from the disproportionality indices reveal the need for targeted interventions and policy adjustments in Milwaukee communities. These indices do not only demonstrate disparities but are also critical for guiding approaches that can address racial imbalances in mental health, steering toward a more equitable health care system. Our findings align with research demonstrating that mental health outcomes are the products of spatially located phenomena, including socioeconomic conditions, access to health care, environmental factors, and deeply rooted SRD [36]. This confluence of factors reinforces the assertion that conventional global “one-size-fits-all” approaches fall short of understanding complex phenomena such as community mental health or the intricacies of structural racism and discrimination [19,23]. When processes are governed by their location and by conditions that fluctuate over time, the use of localized spatial modeling approaches offers a more realistic alignment, yielding more accurate and nuanced results. This perspective challenges traditional frameworks and calls for a tailored approach that considers the unique characteristics of each community, reflecting a more comprehensive understanding of mental health disparities (Table 1) [37-39].

While our approach introduces innovative methods for analyzing mental health disparities, it is crucial to acknowledge the inherent limitations of observational studies. Such limitations highlight the need for caution in interpreting causality from our findings. Our study’s results, therefore, should be considered indicative of associations rather than definitive causal relationships. For example, the relationships of several factors with mental health (eg, smoking, sleep deprivation, and unemployment) are likely bidirectional. This acknowledgment is vital in guiding future research and formulating public health policies that are based on a comprehensive understanding of mental health determinants.

Finally, while our study demonstrates that Black residents are overrepresented in neighborhoods where high-risk factors for poor mental health are localized, our approach does not address risk for Black community members who live in non-Black majority communities. Moreover, our classification of communities as Black, Hispanic, and White does not recognize diversity within these racial demographics (eg, Puerto Rican vs Mexican heritage) [40].

### Conclusions

Understanding the complex interplay between SRD and mental health is vital to informing public health policies and interventions. For example, acknowledging that SRD places additional burdens on an individual’s ability to cope with life’s demands calls for targeted support in areas such as mental health services, education, employment, and community infrastructure. Our study, deploying advanced Geographic Information System and unsupervised machine learning analyses, unravels complex spatiotemporal relationships predicting poor mental health while excluding explicit race-related variables. The findings highlight that the risk for poor mental health is intertwined with structural and spatially localized factors that correspond with disproportional racial representation within communities. These insights illustrate a need for reinvestment strategies that recognize, protect, and promote mental health, with a focus on communities disproportionately affected by SRD. Such strategies must be implemented in a manner that considers the multifaceted risks and includes protections against further exacerbating disparities. In an era where mental health disparities persist, our research emphasizes the importance of a targeted and localized approach, prioritizing communities with historical burdens of discrimination, ensuring equitable access to resources, and ultimately fostering a more resilient and inclusive mental health care system.

## Appendix

Multimedia Appendix 1 Geographic variation in place-based risk factors impacting mental health. Maps represent a visualization of the localized influences of 6 place-based risk factors—prevalence of smoking, lack of health insurance, poverty level, insufficient sleep, employment status, and median age—on predictions of poor mental health. Visualization is based on the findings of the GWRF model. Each risk factor is represented in a separate map, with color gradients signifying areas of varying impact on mental health. Darker shades indicate regions where a risk factor significantly correlates with poor mental health, while lighter shades correspond to weaker correlations. These visual representations underscore the spatial heterogeneity of mental health determinants and the significance of targeted interventions. GWRF: geographically weighted random forest.

## Abbreviations

GWRF: geographically weighted random forest
HIPAA: Health Insurance Portability and Accountability Act
NIMHD: National Institute on Minority Health and Health Disparities
OOB: out-of-bag
SOM: self-organizing map
SRD: structural racism and discrimination
VIF: variance inflation factor
VSURF: Variable Selection Using Random Forest

## Data Availability

The data sets used in this study are publicly available and were sourced from the Centers for Disease Control and Prevention’s 500 Cities Project and the United States Census Bureau.
