# Supplementary figures and images for: Exploring the Association Between Structural Racism and Mental Health: Geospatial and Machine Learning Analysis
Source: JMIR Public Health Surveill. 2024 May 3;10:e52691. doi: 10.2196/52691 (PMC11102033; doi:10.2196/52691)

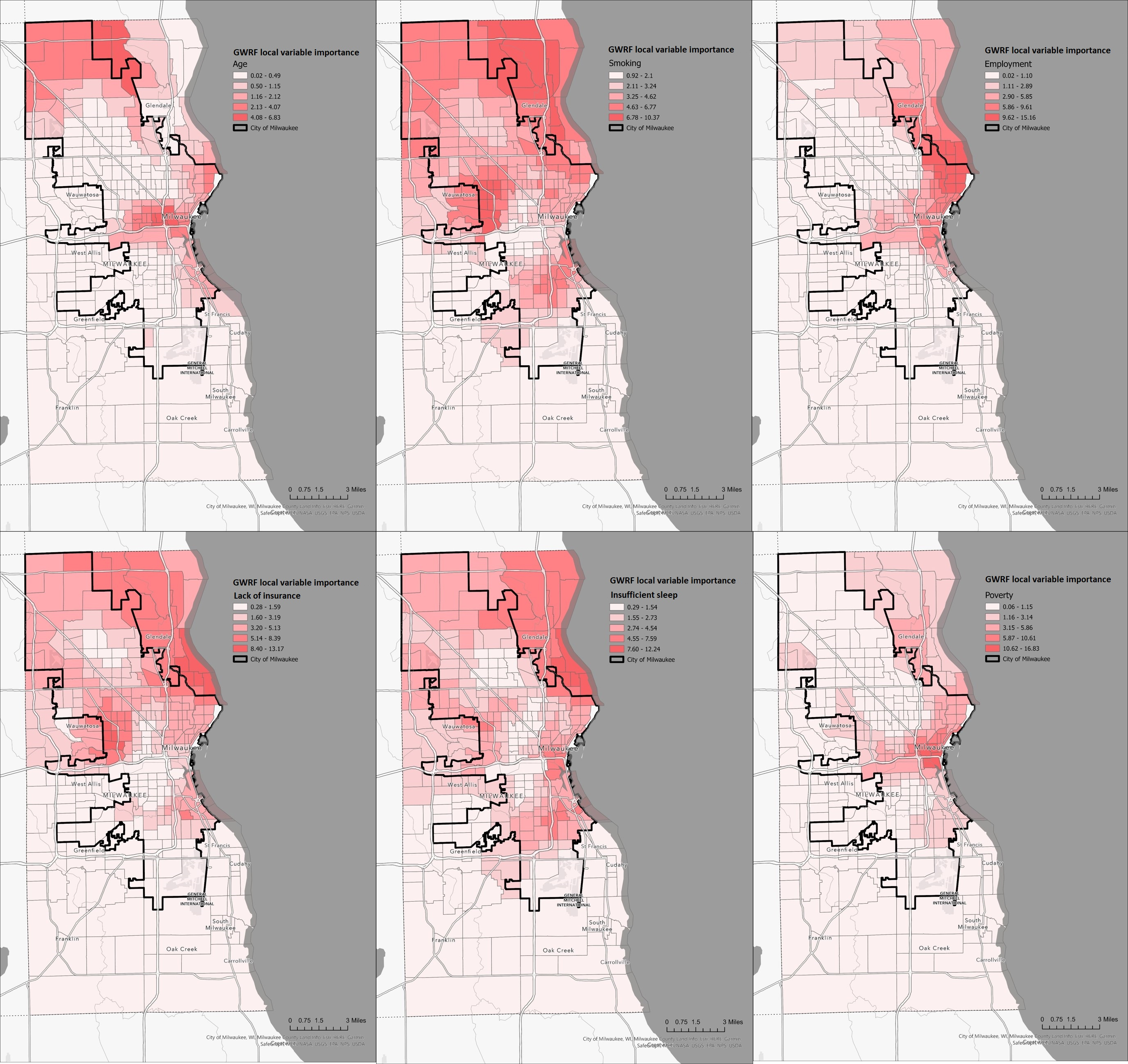

Supplement: Multimedia Appendix 1 [file publichealth_v10i1e52691_app1.png]
